# Barriers and facilitators influencing access to and utilization of primary healthcare services in Kurdistan-region, Iraq: a cross-sectional study

**DOI:** 10.1097/MS9.0000000000000957

**Published:** 2023-06-10

**Authors:** Kochr Ali Mahmood, Abubakir Majeed Saleh

**Affiliations:** a Department of Community Medicine, Hawler Medical University; b Department of Medicine, University of Koya, Koya; c Department of Nursing, Tishk International University, Erbil, Kurdistan-region, Iraq

**Keywords:** barriers, PHC centres in Erbil governorate, PHC centres, primary healthcare services, quality of PHC in Kurdistan-region

## Abstract

The primary healthcare (PHC) idea is intended to become the first line of interaction for members of the public with care needs, as well as a system that views health as a complete state of being rather than only a state of wellbeing. This study aimed to examine the barriers and facilitators influencing the access and utilization of PHC services in Erbil governorate, Kurdistan-region, Iraq, through assessing populations’ practice, and satisfaction. Also, explore the relationship between the socioeconomic, demographic, and cultural characteristics of the study population and utilization of PHC services.

**Methods:**

This was a cross-sectional study. A questionnaire-based survey was used for data collection. Totally, 2400 individuals have been selected in 6 different districts and the centre of Erbil through the multi-cluster random sampling method. The χ^2^ test was used for categorical variables, and a one-way ANOVA was used for numerical variables. A *P* value less than 0.05 was considered statistically significant.

**Results:**

The main reason for utilizing PHC centres was preventive purpose (68.1%), then poverty was the second reason (11.33%), and the participants reported that during the presence of urgent cases when they cannot use other health facilities, they use PHC centres (9%). In terms of barriers for utilizing and visiting PHC centres, the participated people stated that most of them, due to inadequate services, did not use and visit PHC centres (83.21%); the second reason was the presence of chronic diseases such as hypertension, which makes them visit private clinics (7.79%) and generally, (31.4%) of the participants were satisfied with the health services nearby.

**Conclusion:**

In conclusion, it appears that many people visit PHC facilities, but most of them only do so as a preventative measure, and very few go there to obtain basic medical treatment. Most patients go to private clinics and/or hospitals since those facilities have better access to specialists, better quality and quantity of medications, and laboratory testing. Additionally, combining and strengthening service quality aspects that prioritize a patient-centred environment and an effective service delivery system is a key strategy for the health sector to increase patient satisfaction.

## Introduction

Highlights

- The Iraqi healthcare system has endured years of turbulence, conflict, mismanagement of funds, and continuous sectarian violence.
- The lack of laboratory tests, the low quality and quantity of available medications, and the dearth of professionals all proved to be formidable obstacles.
- A total of 2400 individuals participated in the current cross-sectional study.
- The main reason for utilizing primary healthcare centres was a preventive one, followed by poverty.
- People’s satisfaction was very low, especially in places without specialized physicians.

Primary healthcare (PHC) is designed to be the primary point of contact for the public with care requirements and a system that regards health as a whole state of being rather than just wellbeing^1^. PHC is also important since it provides essential therapeutic and preventative healthcare services to individuals without alternative options^2^. A strong system of healthcare is essential to national health and health equity^3^. Well-coordinated PHC services can improve community health^4^. The Sustainable Development Goals (SDGs) priorities universal, equal access to and PHC service systems^5^.

Healthcare is geographically unequal. PHC centres provide primary and preventative care. PHC centres and public hospitals provide low-cost public healthcare. Most people live in cities, whereas rural areas have secondary centres^6,7^. Poor organization, staffing shortages, and prescription shortages restrict PHC centre services. PHCs are still considered crucial healthcare facilities, especially for the poor people^6,8^. A recent survey found Saudi patients satisfied with primary care^9^. Pakistan has gender discrepancies in PHC access and several challenges to accessing PHC services, including location, distance, transportation, staff availability, income, service hours, and service organization^10^. In addition, an Iraqi assessment found that infrastructure was adequate, but human resources and equipment and supplies were scarce. However, doctors and patients agreed it fell short^11^.

Iraq’s public health system, including Kurdistan, includes primary care and secondary and tertiary hospitals. The former has population specific PHC centres. Half are physician-staffed main centres and half are nurse-staffed or medical assistant-staffed smaller centres^12^.

Iraq’s healthcare system has suffered from political and socioeconomic events over the previous few decades, resulting in a reduction in major health indices and a crippled system unable to meet population needs. The primary care system was devastated and still struggles with healthcare system challenges^13–15^.

The Kurdistan Region Ministry of Health has improved healthcare services, including PHC and chronic disease centres, but there is little research on population PHC utilization. A few years ago, a team of experts pointed out the substantial gaps in PHC services in Kurdistan, including a lack of doctors, nurses, and equipment at primary care centres^16^. Despite a lack of scientific proof, numerous media outlets have claimed that PHC facilities are deficient, causing a reduction in visitors. However, Erbil City, Iraq, patients were satisfied with private clinics but not governmental hospitals^17^. This study assessed population practice and satisfaction to identify barriers and facilitators to PHC service use in Erbil governorate, Kurdistan Region of Iraq. Also examined PHC use and socioeconomic, demographic, and cultural features of the research group.

## Methods

### Study design

Cross-sectional study.

### Setting

The study randomly selected a cluster of six households from the catchment areas of 75 main health centres, which are all doctor-staffed PHC facilities in Erbil governorate.

### Period of the study

This study was conducted between 16 August 2021 and 20 February 2022 in Erbil governorate, Kurdistan Region of Iraq.

### Sampling technique and procedures

**Figure 1:**
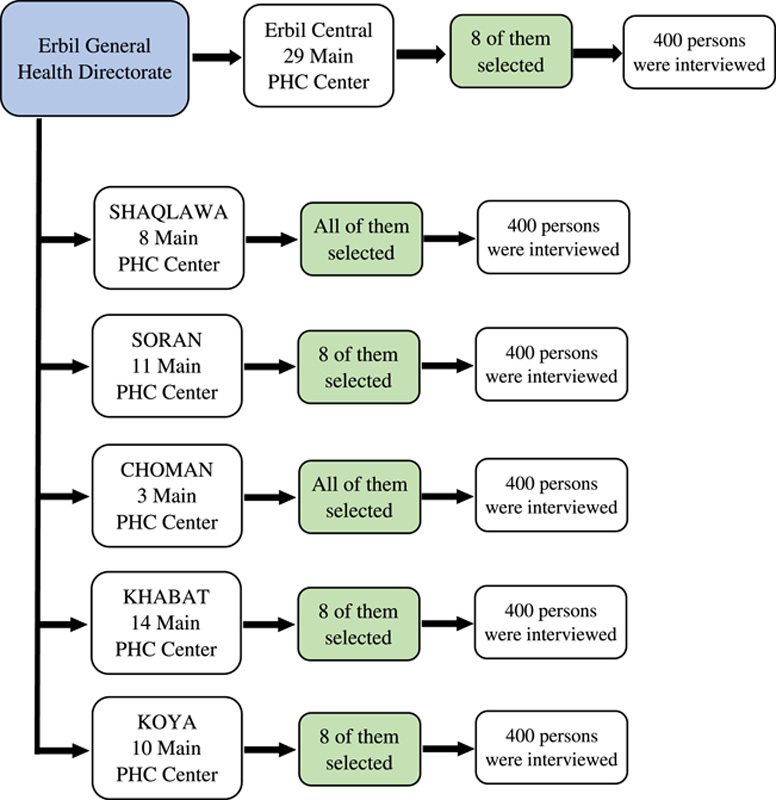
Describes the process of sampling techniques. PHC, primary healthcare.

A cluster sampling was used firstly to select PHC centres in six districts of Erbil governorate. This governorate including six different districts (centre of Erbil, Choman, Soran, Shaqlawa, Khabat, and Koya”koe sinjaq”). Then simple random sampling was used to select individuals to answer the questionnaire (Fig. 1). The homes located within 2 km of a PHC facility were its catchment or service area. Streets were arranged in practically every location in a grid pattern. Due to the absence of GPS, a housing selection technique that first chooses a random route from the medical centre was implemented. The first house was selected by throwing a pen and then taking other samples were completed with the next door. The process was repeated if the chosen direction did not point in the direction of a residential neighbourhood.

### Inclusion and exclusion criteria

Once the sample had been chosen, every adult over the age of 18 and under the age of 70 were included, while unhealthy people and above 70 years were excluded in the study.

### Sample size

Through using the Epi Info program version 7.2, based on the default value of population size, an expected rate of 50% and a confidence interval of 95%. The estimated general population in Erbil governorate, including all other districts, is 1 530 722^18^. For the six places, ~384 people should be interviewed, while the number was increased to 400 in each place. That means a total of 2400 adult people are included in the current study.

### Study tools and measures

A structured questionnaire was used to obtain information on the demographic, socio-cultural, and economic characteristics of the users; their use of health services; different barriers to utilizing health services, and their satisfaction with the PHC services. The research questionnaire was developed based on the previously published studies^11,19–21^. The questionnaire was translated to Kurdish language by linguistic experts. A forward-backwards translation from English to Kurdish ensured precise results (Supplementary file 1). Before conducting the study, to know the internal validity, the questionnaire was sent to ten specialist and experts in community medicine field. They had their comments, and all the comments were considered to change some questions. Then 30 samples were taken from the same population to know the reliability. The Cronbach alpha was 0.81 for the whole questionnaires. For satisfaction, 14 questions were asked, answered by strongly dissatisfied to strongly satisfied Each Satisfaction item received the following scores: strongly disagree and strongly disagree equals −1, don’t know equals zero, and satisfied with highly satisfied equals 1. subsequently, to determine overall satisfaction, all the results were collected and divided by the number of questions, 14^17,21,22^.

### Data quality control

The primary author and a group of three nursing professionals with bachelor’s degrees gathered the data. A 3-day training session was conducted to educate the data collectors and supervisor on the objectives of the study, the contents of the questionnaire, and the various interviewing techniques. The questionnaire in English was prepared. The data were obtained via a personal interview (face-to-face) conducted in person. The trained team entered all collected data using SPSS version 25 simultaneously after collection for each location. The supervisor and principal investigative personnel provided ongoing monitoring and oversight to ensure the thoroughness and coherence of the collected data.

### Ethical consideration

The study was conducted in line with the Helsinki Declaration. The ethical approval was given by the college of medicine at Hawler Medical University with reference number (2/1465) on 17/5/2021. The written inform consent was obtained from all participants. The present study adheres to the STROCSS 2021 criteria^23^.

### Data analysis

All data analysis were performed using IBM SPSS Statistics version 25 (IBM Corporation). A simple table was used to explain qualitative information and mean ± S.D was used to quantify variables. To examine the associations, the χ^2^ test was used for categorical variables and one-way ANOVA was used for numeric variables, after changing the answers for mean score, they were collected then compared in each place. In addition, a *P* value less than 0.05 was considered statistically significant. All the analysis was done by the statistical package for social science (SPSS version 25). Also, graphpad prism 8 was used to create graphs.

## Results

In total, 2400 people were included in this study. The mean age ± SD was (40.02 ± 12.68). Most of the participants were female (54.8%). In terms of the education level, most of them graduated from primary school. More than half reported that their monthly income is enough for daily needs (63.9%). The proportion of married people was 77.3%, as well as (39.8%) were housewives. Additionally, people from sub-urban areas made up the largest percentage of those who participated in the study (63.9%) (Table 1).

**Table 1:** Sociodemographic characteristics of the participants

Variables	Frequency, *n* (%)
Mean Age ± SD	40.02 ± 12.68
Sex	
Female	1316 (54.8)
Male	1084 (45.2)
Level of education	
Illiterate	681 (28.4)
Read and write	313 (13)
Primary	700 (29.3)
Secondary	198 (8.3)
Institute	283 (11.8)
Collage	200 (8.3)
Post-graduate	25 (1)
Monthly income	
Not enough	441 (18.4)
Enough for daily needs	1534 (63.9)
Exceeds needs	427 (17.8)
Marital status	
Single	405 (16.9)
Married	1856 (77.3)
Widow	131 (5.5)
Divorced	8 (0.3)
Occupation	
Non-employee	152 (6.3)
Self-employee	415 (17.3)
Governmental employee	658 (27.4)
Retired	62 (2.6)
Student	159 (6.6)
House wife	954 (39.8)
Place of residency	
Urban	402 (16.8)
Sub-urban	1534 (63.9)
Rural	464 (19.3)

**Figure 2:**
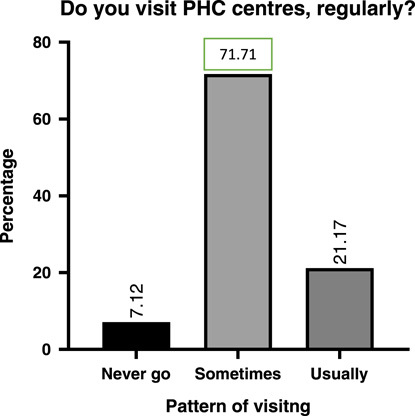
Shows visiting PHC centres. PHC, primary healthcare.

**Figure 3:**
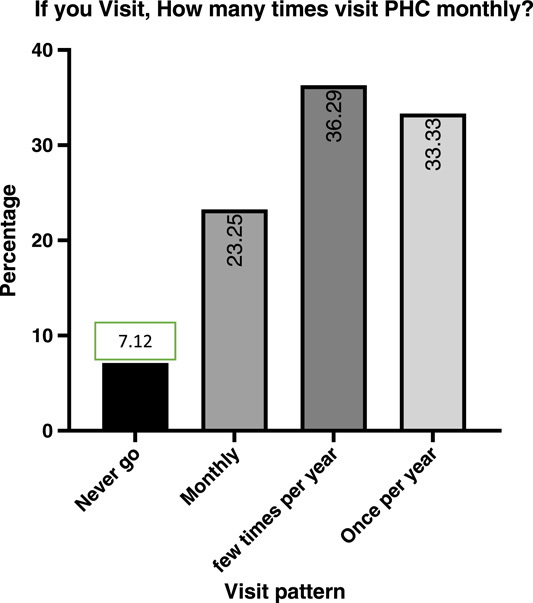
Shows the patterns of visiting PHC centres, Monthly. PHC, primary healthcare.

**Figure 4:**
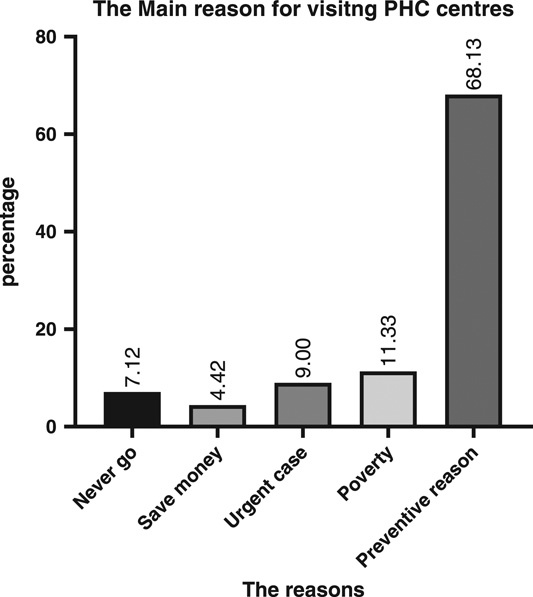
Shows the main reasons for visiting PHC centres. PHC, primary healthcare.

Regarding visiting and utilizing PHC centres, most of the participants visit there sometimes (71.7%), while only (7.1%) reported that they never utilize PHC centres. In addition, among those who visit there, most of them use them a few times annually (36.29%), while (33.33%) of the participants visit there once per year, and only (23.25%) utilize the PHC centre monthly. (Figs. 2 and 3). In addition, the main reason for utilizing was a preventive purpose (68.1%), then poverty was the second reason for visiting (11.33%), and the participants reported that during the presence of urgent cases when they cannot use other places, they use PHC centres (9%) (Fig. 4). Furthermore, in the details of the reasons for their visits, they reported that (89.3%) of them visit to take care of their pregnancy and children’s healthcare, while (49.4%) use them for vaccination, and (30.2%) use them for curative purposes and to receive very simple treatment (Table 2).

**Table 2:** Practice of the participants regarding utilizing PHC centres

Why you visit PHC centres?	Frequency, *n* (%)
Maternal care	2143 (89.3)
Vaccination	1185 (49.4)
Malnutrition	77 (3.2)
Dental care	839 (35)
Health education	4 (0.2)
Chronic diseases	113 (4.7)
Rehabilitation purpose	49 (2)
Ultra sound and x-ray	725 (30.2)
Curative purpose (very simple medication)	442 (18.4)
Visit for license and lab. purpose	759 (31.6)
Other reason	9 (0.4)

PHC, primary healthcare.

**Figure 5:**
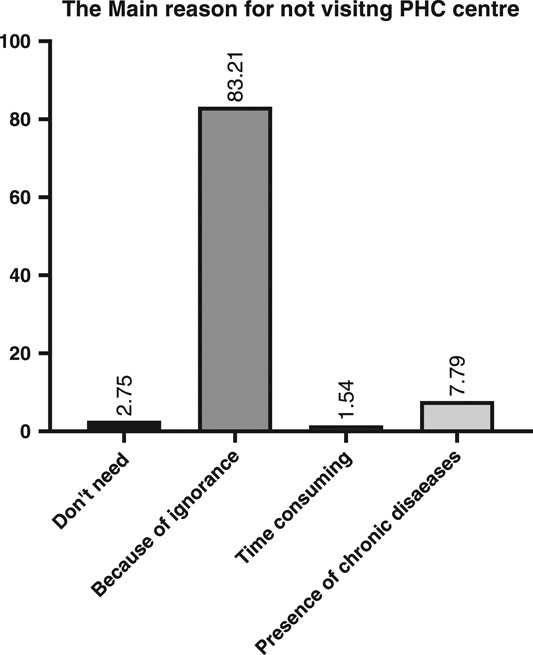
Shows the main reasons for not visiting PHC centres. PHC, primary healthcare.

In terms of barriers for utilizing and visiting PHC centres, the participated people stated that the majority of them, due to inadequate services, did not use and visit PHC centres (83.21%); the second reason was the presence of chronic diseases such as hypertension, which makes them visit private clinics (7.79%); and finally, a few people stated that they do not need to visit there (2.75%) (Fig. 5). Furthermore, when the details were asked, the results indicated that many people do not visit there due to a lack of specialist physicians (92.3). Moreover, 95.8% indicated that they go to hospitals or private clinics instead of PHC centres, and 85.6% stated that in the laboratory there are not adequate materials to investigate their health problems. Furthermore, 50.6% reported a low quantity of medications inside PHC centres, and 49.4% reported a low quality of medications (Table 3).

**Table 3:** Barriers of visiting people to PHC centres

Explain more reasons for not going to PHC	Frequency, *n* (%)
Language barriers	21 (0.9)
Using private clinics and hospitals	2298 (95.8)
I don’t trust PHC services	430 (17.9)
Long distance	50 (2.1)
Low quality of medications	1186 (49.4)
Low quantity of medications	1215 (50.6)
Bad behaviours	97 (4)
Long waiting time	65 (2.7)
Unaffordable	26 (1.1)
Lack of specialist	2214 (92.3)
Stigma	28 (1.2)
I don’t have time in the morning	33 (1.4)
Working hours is short	102 (4.3)
Physicians stay for short period of time	65 (2.7)
Medications are available for short period	87 (3.6)
Lack of laboratory tests	2054 (85.6)
Other reasons	168 (7)

PHC, primary healthcare.

The satisfaction of the participants was different. (27% and 28.1% of them were strongly dissatisfied and dissatisfied, respectively, with the services in PHC centres). While, for other questions they were strongly satisfied or just satisfied, such as: satisfaction with nurses; satisfied with time spent with patients; satisfied with treatment and care received; satisfied with accessibility of healthcare services; satisfied with accessibility of healthcare services; the physician’s humanity is good; satisfied with the PHC environment; satisfied with the waiting time; satisfied with crowding; satisfied with the preventive program; satisfied with the administration staff; satisfied with communication between health and medical staff; satisfied with treatment and diagnosis costs). Furthermore, approximately (28%) were both strongly dissatisfied and strongly satisfied with the presence of physicians. Generally, (31.4%) of the participants were satisfied with the health services nearby (Table 4). As well as, in terms of satisfaction in different places, Koya City had the worst satisfaction compared with other cities, and all the associations were statistically significant (Table 5).

**Table 4:** Satisfaction of the participants regarding PHC services

Questions	Strongly dissatisfied, *n* (%)	Dissatisfied, *n* (%)	Don’t Know, *n* (%)	Satisfied, *n* (%)	Strongly satisfied, *n* (%)
To what extend you satisfied with the health services in PHC generally	647 (27)	675 (28.1)	15 (0.6)	487 (20.3)	576 (24)
Satisfaction with nurses	260 (10.8)	346 (14.4)	21 (0.9)	844 (35.2)	929 (38.7)
Satisfied with time spend with patients	477 (19.9)	528 (22)	32 (1.3)	629 (26.2)	734 (30.6)
Satisfied with treatment and care received	501 (20.9)	542 (22.6)	25 (1)	660 (27.5)	672 (28)
Satisfied with accessibility of healthcare services	426 (17.8)	459 (19.1)	27 (1.1)	774 (32.3)	714 (29.8)
Satisfaction with presence of physicians	688 (28.7)	406 (16.9)	43 (1.8)	581 (24.2)	682 (28.4)
The physician’s humanity is good	198 (8.3)	341 (14.2)	17 (0.7)	845 (35.2)	999 (41.6)
Satisfied with the PHC environment	252 (10.5)	197 (8.2)	29 (1.2)	681 (28.4)	1241 (51.7)
Satisfied with the waiting time	302 (12.6)	326 (13.6)	35 (1.5)	614 (25.6)	1123 (46.8)
Satisfied with crowding	324 (13.5)	334 (13.9)	23 (1)	601 (25)	1118 (46.6)
Satisfied with the preventive program	156 (6.5)	219 (9.1)	64 (2.7)	582 (24.3)	1379 (57.5)
Satisfied with the administration staff	258 (10.8)	227 (9.5)	317 (13.2)	408 (17)	1190 (49.6)
Satisfied with communication between health and medical staff	101 (4.2)	157 (6.5)	135 (5.6)	478 (19.9)	1529 (63.7)
Satisfied with treatment and diagnosis costs	88 (3.7)	23 (1)	23 (1)	204 (8.5)	2062 (85.9)
Overall satisfaction	Frequency, *n* (%)				
Satisfied	754 (31.4)				
Neutral	1037 (43.2)				
Unsatisfied	609 (25.4)				

PHC, primary healthcare.

**Table 5:** Association between satisfaction and cities^a^

		95% CI		
City	Mean difference	Upper	Lower	P*[Table-fn T5fn2]
Erbil	−4.6	−6.5	−2.7	<0.001
Khabat	−11.14	−13.02	−9.26	<0.001
Shaqlawa	−10.9	−12.8	−9.1	<0.001
Soran	−3.01	−4.8	−1.13	0.02
Choman	−5.8	−7.7	−3.9	<0.001

a Koya is based.

* Taken from ANOVA.

Both males and females were satisfied equally since the association was not significant statistically. People who had a higher education level were less satisfied, and this relationship was significant. In addition, the participants with not enough monthly income were less satisfied compared with others. As well as rural areas, the population had the highest satisfaction percentage compared with other areas. Regarding occupation, housewives had the majority of very satisfied. Further, married people were more very satisfied than others. All the associations were statistically significant (Table 6).

**Table 6:** Association between satisfaction level and demographic characteristics

	Satisfaction level			
Variables	Poor (%)	Satisfied (%)	Very satisfied (%)	*P*
Sex				0.9
Male	26.8	29.1	44.2	
Female	26.4	29.5	44.1	
Level of education				<0.001
Illiterate	20.9	29.1	50.1	
Read and write	29.7	35.8	34.5	
Primary	23.3	28.4	48.3	
Secondary	35.9	23.2	40.9	
Institute	33.6	31.1	35.3	
Collage	32	23	45	
Post-graduate	40	56	4	
Monthly income				<0.001
Not enough	22.7	39	38.3	
Enough for daily needs	26.4	28.6	45	
Exceeds needs	31.1	21.8	47.1	
Place of residency				<0.001
Urban	35.8	22.4	41.8	
Sub-urban	27.6	29.8	42.6	
Rural	15.3	33.6	51.1	
Occupation				<0.001
Non-employee	36.8	30.9	32.2	
Self-employee	31.1	26.7	42.2	
Governmental employee	27.2	27.1	45.7	
Retired	21	43.5	35.5	
Student	29.6	25.2	45.3	
House wife	22.4	31.4	46.1	
Marital status				<0.001
Single	32.3	35.1	32.6	
Married	24.1	28.6	47.4	
Widow	39.7	23.7	36.6	
Divorced	100	0	0	

In terms of utilizing PHC centres, females usually go there with a percentage of 23.6%, while males go there with a percentage of 18.2%. Further, people who had less level of education used PHC centres compared with people who had more level of education. In addition, poor people who do not have enough monthly income as well as those who live in rural areas lose more compared with others. Additionally, housewives and married women usually visit PHC centres more often than other workers. All the relationships were statistically significant (Table 7).

**Table 7:** Association between practice and demographic characteristics

	Going to PHC centres			
Variables	Never go (%)	Sometimes (%)	Usually go (%)	*P*
Sex				0.005
Male	7.5	74.4	18.2	
Female	6.8	69.5	23.6	
Level of education				<0.001
Illiterate	3.4	70.2	26.4	
Read and write	5.4	69.6	24.9	
Primary	8.7	72.4	18.9	
Secondary	11.6	71.7	16.7	
Institute	8.1	77	14.8	
Collage	10	69.5	20.5	
Post-graduate	16	76	8	
Monthly income				<0.001
Not enough	6.8	66.7	26.5	
Enough for daily needs	7.3	70.8	21.9	
Exceeds needs	6.8	80.1	13.1	
Place of residency				<0.001
Urban	11.4	66.7	21.9	
Sub-urban	7.2	72.4	20.4	
Rural	3.2	37.7	23.1	
Occupation				<0.001
Non-employee	11.8	77.6	10.5	
Self-employee	8	72	20	
Governmental employee	10.6	68.7	20.7	
Retired	12.9	75.8	11.3	
Student	5.7	81.8	12.6	
House wife	3.5	70.8	25.8	
Marital status				<0.001
Single	9.4	78.8	11.9	
Married	5.8	70.3	24	
Widow	19.8	68.7	11.5	
Divorced	0	100	0	

PHC, primary healthcare.

## Discussion

### Main findings

It was clear that, the participants visit PHC facilities for preventive reasons such as maternal and child healthcare, vaccinations, and laboratory tests for work-related purposes. While the lack of proper health services in PHC clinics was the main deterrent, additionally, the bulk of people go to private or hospital clinics. Other factors were discussed, including a shortage of specialists, poor medicine, especially regarding quality and quantity, and insufficient laboratory tests and investigations conducted inside PHC facilities. Finally, approximately half of the participants were neutral with the PHC facilities’ services, although Koya residents were less satisfied than participants from other locations.

### Compare with other studies

Regarding practice and visit PHC centres, many people reported that, they visit PHC centre while the preventive aspect was the main reason. This was compatible with another study which concluded that most people preferred private health facilities rather than public sectore^6,24^. In addition, PHC centres are visited more frequently by individuals who have chronic diseases as opposed to regular people or those who have acute or straightforward illnesses^25^. In the current study it is found that females visit PHC more than males, this was proved in other study in Oman^26^. While, in another Asian country, although though Haiti had a large network of health facilities, only a small percentage of the population, particularly in rural regions, had access to primary care facilities that met acceptable standards of excellence^27^.

In general, Iraq’s healthcare system has suffered from many years of turmoil, war, misuse of funding, and ongoing sectarian bloodshed. Iraq is now undergoing continuing reconstruction efforts. The difficulties include an educational system with low enrolment and instructors who are equipped to teach the curriculum. There is a severe lack of modern healthcare facilities, tools, and staff, which has caused death rates to rise everywhere ^28^. Because of this reason many people do not trust health system in Iraq and Kurdistan-region^17^.

Consumer satisfaction refers to the degree of happiness experienced by customers after using a service. It illustrates the discrepancy between the standard of service and the client’s experience with that standard. Consumer or patient satisfaction measurements are becoming an essential component of global hospital or clinic management strategies. Additionally, the majority of nations’ quality assurance and certification procedures call for frequent measurements of customer satisfaction^29^. As a result, delivering recognized standards of care now includes one of the key factors: consumer satisfaction with health services. As it is essential for clients to use services, adhere to treatments, and maintain a long-term connection with practitioners, satisfaction has been considered to be a primary predictor of usage of services^30^. A high overall satisfaction rating with PHC services is commendable and can be viewed as a significant indicator of the quality of healthcare services to forecast both capacity and consumption that are connected to the consistency of care, the communication skills of the medical professionals, and the public’s trust in the healthcare system^31–33^.

Previously, people’s satisfaction in Iraq for health services was good, and many people stated that they were satisfied with the PHC services, a study in Al-Ramadi City, West Iraq, revealed that about 47% of patients expressed positive views and general satisfaction towards all services provided, with the highest proportion (64.8%) being satisfied with the cost of services being affordable and the lowest proportion (13.7%) being satisfied with the availability of doctors in the clinic^34^. ‬‬‬ Studies conducted in other nations revealed that patients and customers of PHC providers in that country had very high levels of contentment with the services provided in their location^35–38^.

On the other hand, another study proved that most of the participants dissatisfied with these services, as revealed in the current study^39^. A quantitative investigation of the present position and causes of dissatisfaction with local healthcare care will not only allow healthcare institutions to recognize problems and improve their services in a targeted manner, but it will also enhance patients’ trust and reliance on local healthcare, thereby further facilitating timely access to care and promoting health for all. This is because patients will be able to see that the medical institutions are able to identify problems and improve their services in a targeted manner^40^. In the current study most respondents were dissatisfied with the general health services nearby their region, this was proved prior research found that factors related to medical services, such as doctor and nurse services, medical equipment, and waiting times, had a substantial impact on patients’ levels of discontent^41–44^. Level of satisfaction can be impacted by socioeconomic level and some other factors, these factors lead to use private clinics and satisfy more with the non-public health sector^17,45^.

In addition, the participants blamed that in the public sector, physicians and health staff did not give them enough time to treat and explain their conditions. This result is similar with a study conducted by Burnham and his friend in Iraq^24^.

However, another study in the Kurdistan-region indicated that the information also pinpointed locations where users should get training and where laboratory, X-ray, and/or other equipment should be fixed or replaced. Most doctors barely spend 3–4 h per day working in the public sector, despite the fact that the necessary workweek is 35 h, and all physicians are paid for these 35 h. The rest of the time was often spent working in the private sector for a much higher wage^46^. This can make a noticeable difference between public and private health sectore, at the same time might affect visiting PHC facilities as well.

Furthermore, one of the barriers was medications due to the substandard quality of the prescriptions that are offered there, while others criticized the number of medications as well. This was documented previously in a study which reported that, regarding their understanding of generic medications, most respondents gave the wrong replies. Only around one-third of the participants were aware that generic medications must adhere to identical safety criteria as brand-name medications, are therapeutically equal to brand-name medications (26.6%) and are as safe as brand-name medications (34.7%). Regarding perspective, many doctors had unfavourable opinions on generic drugs, believing that they were of lesser quality (57.3%) and more likely to induce adverse effects (41.1%) than brand-name drugs^47^. Additionally, another study revealed that, the availability of medicines was the subject of many respondents’ primary concerns^48^. This problem has arisen in other countries in the past; for instance, the quality of diagnosis and treatment was poor in PHC institutions in China; issues such as excessive use of antibiotics, inadequate treatment of noncommunicable diseases, and poor management of chronic diseases were prevalent^49–51^.

Also, up until 2004, the primary importer and distributor of medications and medical supplies for the entirety of Iraq was Kimadia, the government-owned provider of these items. It now solely serves the public sector. The budget for Kimadia was $1.25 billion in 2011. The Kurdish Regional Government receives 17% of all purchases, while the rest of Iraq receives 83%. In healthcare institutions, there are frequently shortages of medications and supplies. Insufficient inventories and delivery delays are caused by issues with drawn-out procurement procedures, complex financial methods, and a high turnover of technical employees^52^.

In PHC centres in Baghdad many people visit them to take treatment or other services, while there was a significant difference among various age groups. They reported that consumers used a variety of services, with vaccination accounting for the biggest number, followed by evaluation and treatment, and services for dental care, childcare, and maternity and child health^53^.

People in the current study gave a variety of major reasons for not using PHC facilities. This result is similar to another study that was conducted among pregnant women in Baghdad City. Their reasons were: desire for private healthcare, difficulty leaving elderly children alone, being alone, high income, the number of children, and a history of past abortions. The distance to the medical facility, the lack of knowledge, and the husband’s lack of consent^54^. A separate section for the elderly without a functioning referral system, a scarcity of drinking water in the PHC clinics, and other problems have also been identified as contributing causes^55^.

### Strengths and limitations of the study

The latest study’s strengths include the ability to generalize its findings to the whole community due to its use of face-to-face interviews and surveys, which prevented recall bias, and, in addition, the explanation of many of the factors that prevent individuals from using PHC centres.

There are several restrictions on a survey of this kind, because of using simple random sampling. Only residences that were closer to the PHC centres by 2 km were considered. Others who live further away from the medical facilities may perceive them differently and use them differently than people who live closer to them. Additionally, we excluded the vast majority of PHC centres run by nurses and medical assistants and only counted those with medical physicians as personnel. Participants may overestimate the value of services out of concern over losing access to them, either personally or because of changes in health policy. Further, given that public facilities are often not located in high-income neighbourhoods, choosing families from the catchment areas of PHC centres may have resulted in an overrepresentation of low-income and middle-income households in the research.

## Conclusion

In conclusion, it seems that many individuals travel to PHC facilities, but many of them do so just for preventative purposes, and only a small number go there to receive simple medical care. Numerous obstacles have been identified; the most significant ones were the shortage of experts, the poor quality and quantity of available pharmaceuticals, and the absence of laboratory tests, which is why the majority of patients seek private clinics and/or hospitals. Additionally, with the exception of Koya, everyone was content with these points. It is important for spending on PHC centres treatments, it might boost the efficacy and efficiency of the whole Kurdistan health system. It is recommended that; further studies are needed to explain more barriers for accessing PHC services. Additionally, the public people need to be educated through the various aspects of media such as using posters. Then the government must provide three important necessities which involve specialists, medications, and laboratory equipment, it may affect more people to visit the PHC centres when they need it. Additionally, combining and strengthening service quality aspects that prioritize a patient-centred environment and an effective service delivery system is a key strategy for the health sector to increase patient satisfaction.

## Ethical approval

The study was conducted in line with the Helsinki Declaration. The ethical approval was given by the college of medicine at Hawler medical university (2/1465) on 17/5/2021. The written inform consent was obtained from all participants.

## Consent

The written inform consent was obtained from all participants.

## Source of funding

This research did not receive any specific grant from funding agencies in the public, commercial, or not-for-profit sectors.

## Author contribution

K.A.M. conceived and designed this paper, and wrote the manuscript. K.A.M. and A.M.S. revised the manuscript. The author(s) read and approved the final manuscript.

## Conflicts of interest disclosure

The authors declare that they have no known competing financial interests or personal relationships that could have appeared to influence the work reported in this paper.

## Research registration unique identifying number (UIN)

Not applicable.

## Data availability statement

The datasets used and analyzed in the current study are not publicly available due to condition on ethical restrictions. The raw data that support the findings of this study are available from the corresponding author upon reasonable request.

## Provenance and peer review

Not commissioned, externally peer-reviewed.

## Acknowledgements

The authors thank all the participants who respect them and answered their questions in all cities and districts in Erbil governorate.
